# As easy as ABC: evaluation of safe sleep initiative on safe sleep compliance in a freestanding pediatric hospital

**DOI:** 10.1186/s40621-019-0205-z

**Published:** 2019-05-29

**Authors:** Traci Leong, Manon Billaud, Maneesha Agarwal, Terri Miller, Terri McFadden, Jonathan Johnson, Sarah Gard Lazarus

**Affiliations:** 1 Rollins School of Public Health, Emory University, Atlanta, GA USA; 2 Georgia Department of Public Health, Atlanta, GA USA; 3 Department of Pediatrics, Emory University School of Medicine, Atlanta, GA USA; 4 Department of Emergency Medicine, Emory University School of Medicine, Atlanta, GA USA; 5 Children’s Healthcare of Atlanta, Atlanta, GA USA; 6 Pediatric Emergency Medicine Associates, Atlanta, GA USA

**Keywords:** Pediatrics, Injury prevention, Infant mortality, Sleep safety, Sudden infant death syndrome, Intervention, Quality improvement

## Abstract

**Background:**

The American Academy of Pediatrics (AAP) recommends the ABCs of safe infant sleep (alone, back, clear crib) to combat the increasing rates of Sudden Unexplained Infant Death (SUID). It is unclear if these recommendations are followed for infants hospitalized in pediatric facilities after the newborn period. The objectives of this study were to assess baseline infant sleep behaviors at a tertiary care freestanding pediatric hospital and to evaluate the effectiveness of a hospital-based infant safe sleep program in improving adherence to safe sleep recommendations.

**Methods:**

A quality improvement program with pre- and post- analyses was performed on a convenience sample of infants < 12-months old utilizing a crib audit tool on two general pediatric inpatient units. The crib audit tool was used before and after the safe sleep program intervention. It recorded the infant’s sleep position, location during sleep, and sleep environment. Interventions included: 1) nursing education, 2) crib cards with a checklist of the ABC’s of safe sleep provided for the cribs of hospitalized infants, and 3) tracking boards to report weekly measured compliance with the ABCs. Chi square analysis was used to compare adherence to recommendations before and after program implementation.

**Results:**

There were 62 cribs included pre-intervention and 90 cribs post-intervention. Overall, there was no significant change in safe sleep positioning (81% to 82%, *p* = 0.97). There was a significant increase in adherence to the safe sleep environment recommendation (3% to 38%, *p* < 0.01). Overall safe sleep, including both position and environment, referred to as ABC compliance, improved from 3% pre-intervention to 34% post-intervention (*p* < 0.01). Only 18% of cribs audited displayed a crib card, demonstrating poor compliance on placement of the cards. There was no significant difference in compliance with safe sleep recommendations between infants with a crib card compared to those without (25% vs. 37%, *p* = 0.51).

**Conclusions:**

Significant improvements were made in sleep environments and overall safe sleep compliance after introduction of crib cards and tracking boards. Most likely the crib auditing process itself and the tracking boards had a larger impact than the crib cards.

## Background

Sudden Unexpected Infant Death (SUID) has become one of the leading causes of unintentional infant death in the United States (U.S.). Defined as the sudden and unexpected death of an infant less than 1 year of age, it is characterized by having no immediate or obvious cause. The three common types of SUID include: 1) sudden infant death syndrome (SIDS), 2) accidental suffocation and strangulation in bed (ASSB) or a sleeping environment, and 3) other deaths with an unknown cause. (Centers for Disease Control and Prevention, 2017; Moon, 2016)

Since the 1990s, there has been a reduction in the rate of SIDS deaths following efforts to promote safe infant sleep practices. Despite the success of these early efforts, infant death due to unknown causes and ASSB rates have started to increase from 1997 to date. (Centers for Disease Control and Prevention, 2017; Moon, 2016; Shapiro-Mendoza, 2017). Although SUID deaths are unpredictable, research provides convincing evidence of its association with infant sleep practices. (Moon, 2016; Erck Lambert et al., 2018) Subsequently, the Back to Sleep campaign was changed to the Safe to Sleep campaign in 2012 to reflect the expanded recommendations that address not only infant sleep position, but also the sleep location and environment. (Moon, 2016; National Institute of Child Health and Human Development/National Institutes of Health Safe to Sleep campaign, 2018) Behaviors like placing infants **A**lone, on their **B**ack and in a **C**rib free of extra items reduce an infant’s SUID risk. Together these three behaviors are referred to as the **ABC**s of safe sleep. (Georgia Department of Public Health, 2016)

Sleep-related infant deaths are particularly problematic in Georgia, where every week three infants die due to sleep-related causes. (Georgia Department of Public Health, 2016) To address this problem, the Georgia Department of Public Health (DPH) introduced the “Georgia Safe to Sleep Campaign” in 2016. This campaign included the Georgia Safe to Sleep Hospital-based Initiative at all 78 birthing hospitals within the state. The campaign included: 1) the adoption of safe infant sleep policies based on the 2011 AAP guidelines to reduce the risk of SIDS and other sleep-related infant deaths; 2) education of staff and caregivers on safe infant sleep recommendations; 3) modeling of safe infant sleep by staff; and 4) provision of take home educational materials to every caregiver, including an infant gown that says “this side up,” a safe sleep book, and a bassinette, for those who qualified financially. (Fitzgerald, 2018; Walcott et al., 2018)

Initially, this statewide initiative did not involve children’s hospitals given its focus on birthing hospitals. However, in October 2017, a tertiary-care freestanding pediatric facility partnered with the DPH to participate in the hospital-based initiative. It was hypothesized that baseline compliance with safe sleep recommendations on the general pediatric floors would be poor; however, there would be significant improvement after an educational initiative including: 1) nursing education; 2) availability of crib cards with safe sleep checklists; and 2) use of a motivational tracking board to show ABC compliance. The objectives of this study were to:

1. Assess baseline infant sleep behaviors at a children’s hospital
2. Evaluate the effectiveness of a quality improvement (QI) initiative involving crib cards, tracking boards, and crib audits in improving adherence to the ABCs of safe sleep

## Methods

### Study design

This is a pre-post study designed to evaluate the effectiveness of a QI program of infant safe sleep practices in a tertiary care children’s hospital on two inpatient general pediatric floors. It was deemed exempt by the hospital’s institutional review board (IRB). The program included: 1) nursing education around best practices for safe infant sleep; 2) availability of crib cards with a checklist of safe sleep practices; and 3) weekly reporting of crib audit data to nurses on recorded safe sleep practices utilizing a tracking board.

The pediatric floors included in this QI program contained 35 beds each, with children ranging in age from 3 days to 21 years. These floors were chosen as they did not have a subspecialty focus (such as gastroenterology, neurology, rehabilitation), and we hoped that the patients on these two floors would be the most comparable to a general pediatric population. Patients who were less than 12 months of age and asleep at the time of the crib audit were included in the study. Potential participants were excluded if they were awake, intubated, had craniofacial anomalies, were requiring non-invasive positive pressure ventilation or high-flow nasal cannula, less than 32 weeks gestation, or required isolette or temperature support. If the child was awake, the investigators returned to the room following the remainder of the crib audits and attempted the audit again.

### Data collection

**Fig. 1:**
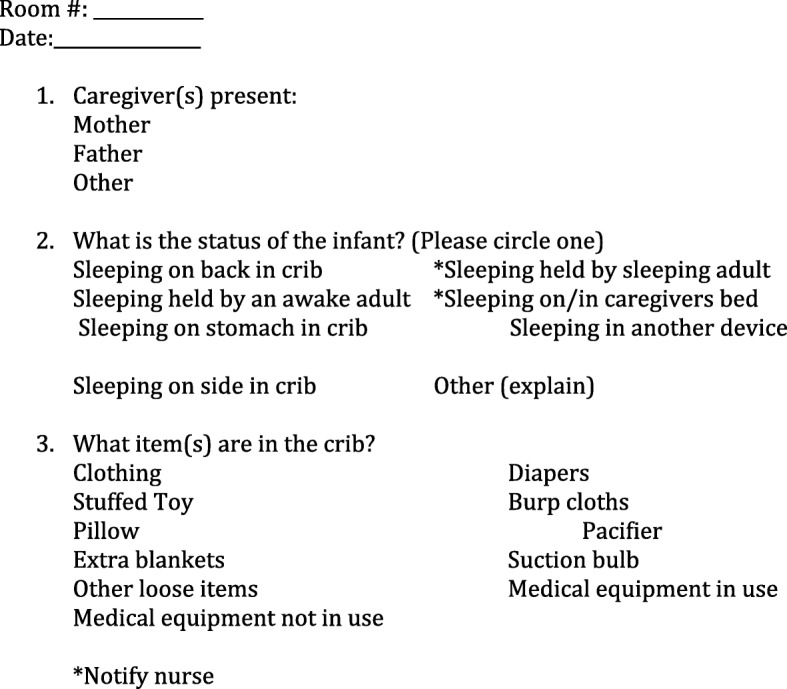
Crib audit tool

A non-random, convenience sampling method was used to identify patients to assess for safe sleep using the Georgia DPH crib audit tool (Fig. 1). This standardized tool is a checklist that records observations on presence of caregiver(s) in the room, sleeping position of the infant, the infant’s location (in crib or on another surface), and item(s) present in the crib’s environment while in the hospital. It can assist in measuring compliance with the safe infant sleep recommendations. This tool had been previously used at all birthing hospitals in the state that participated in the DPH hospital-based safe sleep initiative to help show whether safe infant sleep was being modeled by staff, for parents.

To determine baseline compliance with the ABCs of safe sleep, floor census was reviewed on each day of data collection to identify hospitalized infants less than 12 months of age on two general non-specialized pediatric floors. A team of graduate students and a healthcare provider conducted the crib audits. Data collection for the baseline safe sleep assessments took place over two separate days in the fall of 2017. Patients that had been audited on a previous day were not excluded on a subsequent day, since there was a new opportunity for safe sleep each day. Post-intervention audits were performed on 5 separate days over a four-week period in the spring of 2018 utilizing the same census review and convenience sampling and were performed on the same two general pediatric floors.

Each room with an infant was evaluated for awake or sleeping status, and if sleeping, the positioning, sleep environment, and caregiver presence was assessed. Infants were considered safe in position if they were supine or held by an awake adult. Other positioning options included prone, held by a sleeping adult, on the caregiver’s bed, or in another device. Head of bed elevation was still considered safe sleep provided the child remained supine, due to current hospital policy.

To evaluate environment, the infant’s sleep area was reviewed for items including: blankets, bulb syringe, diapers, clothing, stuffed toys, etc. Safe crib environment was defined as no extra items in the crib except for a pacifier, medical equipment in use, and/or a single swaddle blanket in use. Any additional items in their crib disqualified them from being considered in a safe sleep environment. They were in total ABC compliance if they were in a safe sleep position and environment.

### Intervention

**Fig. 2:**
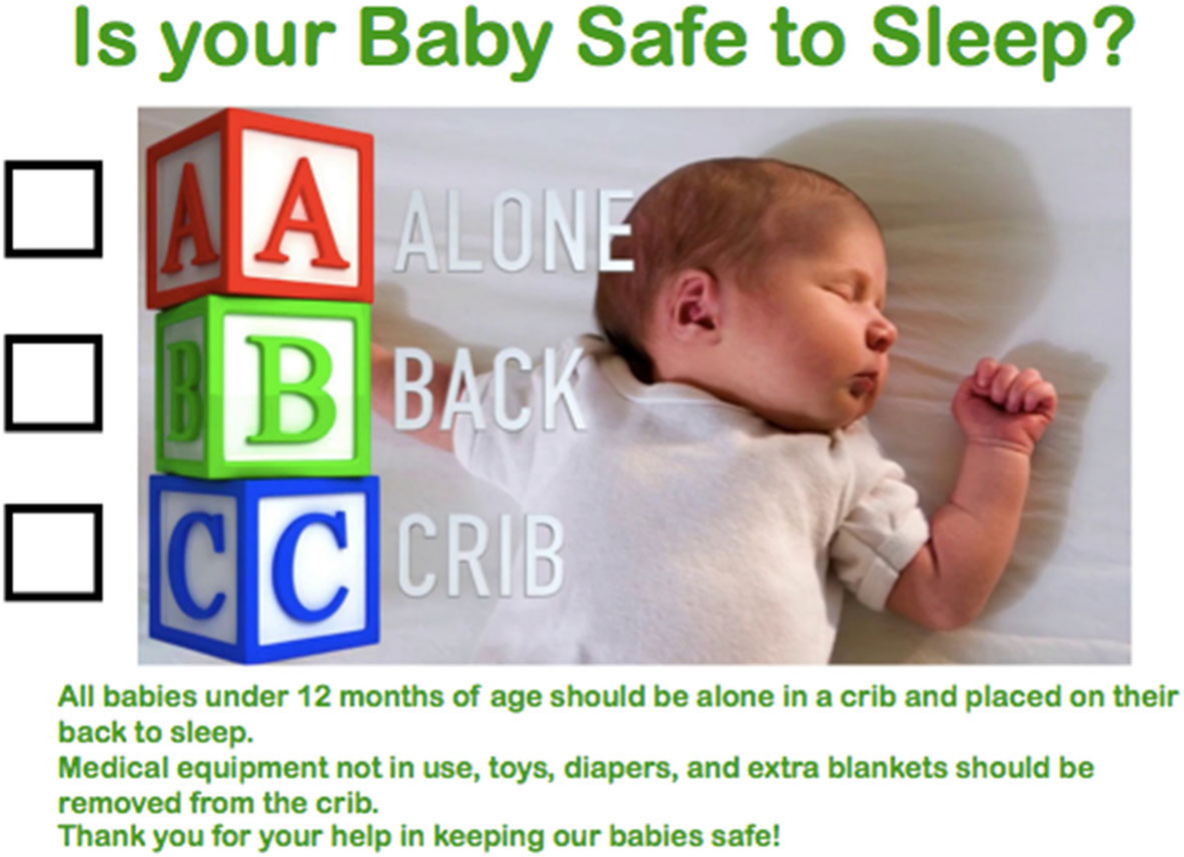
Crib card

Following the results of this pre-intervention evaluation, crib cards with checklists of the ABCs of safe sleep were created based on the most common errors made in positioning and environment (Fig. 2). Collaborative meetings were held with nurse managers on the two included pediatric floors. The crib cards were copied and laminated. Then nurses were asked to apply the crib cards to the slats of the cribs with binder clips to every crib for an infant under 12 months of age. The nurse manager and charge nurses gave reminders during morning huddles to apply crib cards and to adhere to the safe sleep guidelines. Safe sleep handouts were available for families. During this time period a safe sleep carnival took place in the hospital providing updated education about safe sleep. Participation in this safe sleep program was incentivized with prizes and continuing education credits given to attending nurses. A raffle was performed at the end of the study program, with the nurses entering their name each time they educated a family regarding safe sleep or changed an infant’s sleep position from unsafe to safe. The goal of the crib cards was to serve as a visual reminder for healthcare providers to place infants in a safe sleep position and provide a safe sleep environment. Extra crib cards were placed at the secretary’s desk in a folder where other materials and handouts (including isolation signs) were kept.

In addition, nurse managers and nurses were also given information regarding their unit’s baseline poor compliance with AAP recommendations via tracking boards, which were placed in each nursing break room. Displayed on the tracking board weekly, was the progress on the number of cribs audited, the proportion of infants in a safe sleep environment, and the proportion of infants with a crib card correctly hung. The tracking boards were intended to encourage staff to improve their compliance by providing regular feedback on performance. Immediately after implementation of tracking boards and crib cards, post-intervention crib audits were performed using the crib audit tool and the same sampling method as for the initial crib audits. A post-intervention nursing survey was performed that assessed nursing attitudes toward the program and barriers to implementing the ABC recommendations.

### Statistical analysis

For both the pre- and post-intervention phases of the crib audits, data were collected using the online database software, REDCap**©**. Descriptive frequencies were calculated (counts and percentages) to assess the safe sleep behaviors for both phases of the crib audits. Chi square analyses (or Fisher’s exact test when appropriate) were used for categorical variables to compare changes between the pre- and post-intervention phases. Three pre- and post-intervention comparisons were conducted using this analysis evaluating: 1) the proportion of infants in safe sleep position, 2) the proportion of infants sleeping alone (without added items) when sleeping in a crib (i.e. not in an awake caregiver’s arms), and 3) the proportion of infants in compliance with both positioning and safe environment (ABC compliance). In addition, post-intervention, we compared cribs that had crib cards and those that did not to determine if ones with crib cards were more likely to be ABC compliant. The significance level was set as a *p*-value of < 0.05. All analyses were conducted using statistical software *R Core Team,* Vienna, Austria.

## Results

### Crib audits

**Fig. 3:**
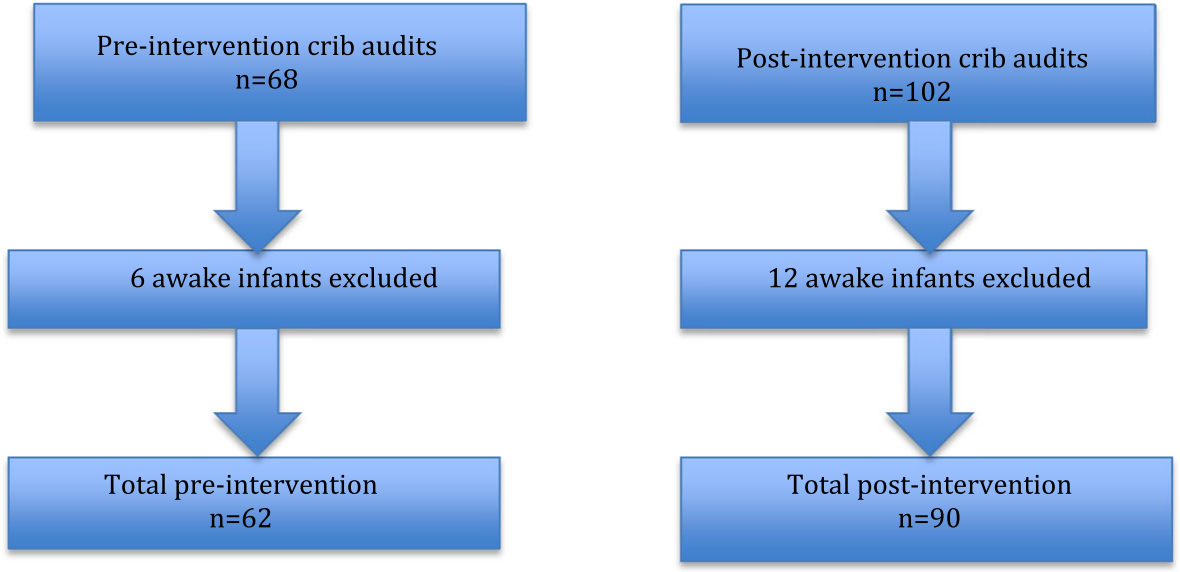
Patient Inclusion flow chart

There were 68 infants screened pre-intervention with 62 (91%) meeting inclusion, and 102 infants screened post-intervention with 90 (90%) meeting inclusion. A total of 170 cribs were screened with 152 infants included in the final analyses. After meeting initial inclusion criteria, four infants were excluded due to their “awake” status (Fig. 3).

### Sleep position

Most infants both prior to intervention (80.6%) and post-intervention (82.2%) were in a safe sleep position (on the back/supine or sleeping in arms of awake caregiver) (Table 1). There was no difference between pre- and post-intervention periods (*p* = 0.97). Unsafe sleep positions and their frequencies are also noted in Table 1. In our study, when including both pre- and post-intervention unsafe sleep position, 9% of infants were prone, 7% were sleeping in a caregivers’ bed, 1% were sleeping held by sleeping adult, and only 2% were side sleeping.

**Table 1:** Evaluation of sleep position pre and post-intervention

Variable	n	Overall (*n* = 152)	Pre (*n* = 62)	Post (*n* = 90)	*p*-value
Safe sleep position, n (%)	152	n (%)	n (%)	n (%)	0.97
No		28 (18.4)	12 (19.4)	16 (17.8)	
Yes		124 (81.6)	50 (80.6)	74 (82.2)	
Sleeping Position, n (%)	152				
Sleeping held by awake adult		39 (25.7)	17 (27.4)	22 (24.4)	
Sleeping held by sleeping adult		1 (0.7)	0 (0.0)	1 (1.1)	
Sleeping on back in crib		82 (53.9)	32 (51.6)	50 (55.6)	
Sleeping on side in crib		3 (2.0)	1 (1.6)	2 (2.2)	
Sleeping on stomach in crib		13 (8.6)	4 (6.5)	9 (10.0)	
Sleeping on/in caregiver’s bed		11 (7.2)	6 (9.7)	5 (5.6)	
Other		3 (2.0)	2 (3.2)	1 (1.1)	

### Sleep environment and ABC compliance

**Fig. 4:**
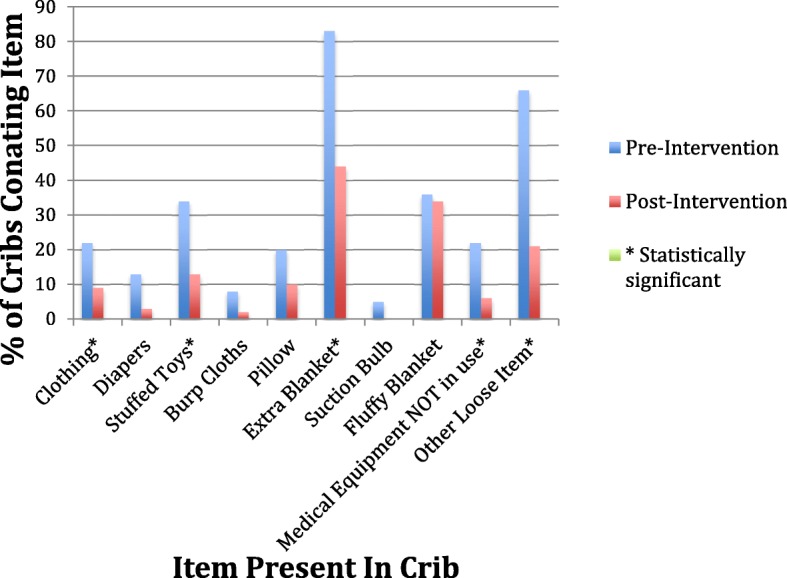
Percent of Unsafe Items Present in Crib Environment Pre and Post-intervention

**Fig. 5:**
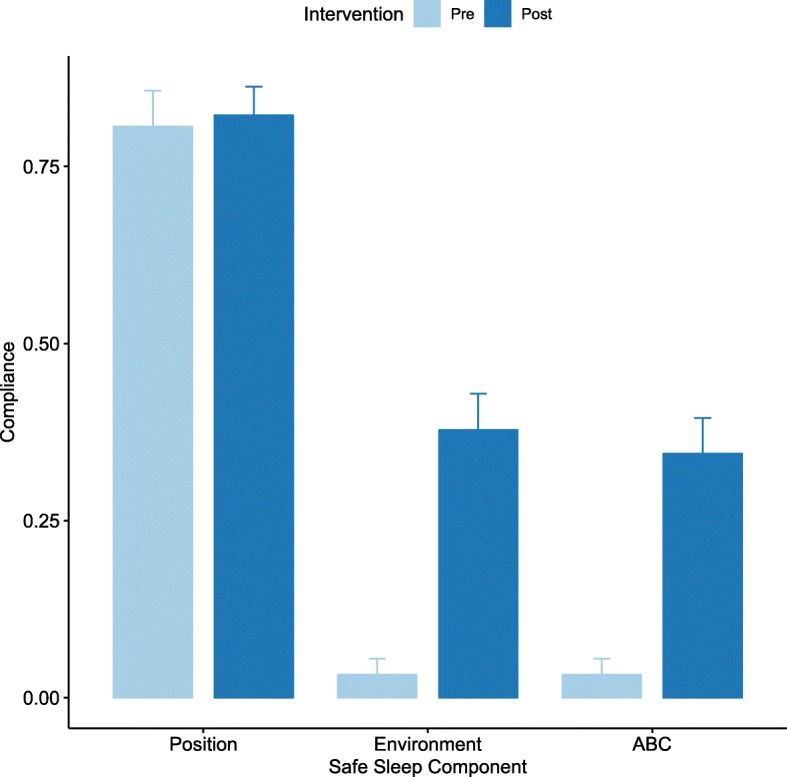
Pre and post-intervention comparisons of sleep position, environment, and overall ABC compliance

The crib environment was significantly safer post intervention (37.8% vs. 3.2%, *p* = < 0.01) (Table 2). The details of the items found in the crib are listed in Table 2. There were statistically significant decreases in the following items found in the cribs post-intervention: clothing (decreased from 22.6% to 8.9%, *p* = 0.03), stuffed toys (decreased from 35.5% to 13.3%, *p* < 0.01), extra blankets (decreased from 82.3% to 44.4%, *p* < 0.01), medical equipment not in use (decreased from 21% to 5.6%, *p* < 0.01), and other loose items (decreased from 64.5% to 21.1%, *p* < 0.01). There were non-statistically significant decreases in all items in the cribs post-intervention, including diapers, burp cloths, pillows, fluffy blankets, and suction bulbs (Fig. 4). Overall ABC compliance significantly improved from 3.2% in the pre-intervention period to 34.4% to the post intervention period (*p* < 0.01) (Table 3). Figure 5 illustrates changes in all three aspects of sleep (positioning, environment, and ABC compliance).

**Table 2:** Safe crib environment

Variable	Overall (*n* = 152)	Pre (*n* = 62)	Post (*n* = 90)	*p*-value
Safe environment, n (%)	n (%)	n (%)	n (%)	< 0.01
Not safe	116 (76.3)	60 (96.8)	56 (62.2)	
Safe	36 (23.7)	2 (3.2)	34 (37.8)	
Clothing, n (%)	22 (14.5)	14 (22.6)	8 (8.9)	0.03
Diapers, n (%)	11 (7.2)	8 (12.9)	3 (3.3)	0.05
Stuffed toy, n (%)	34 (22.4)	22 (35.5)	12 (13.3)	< 0.01
Burping cloths, n (%)	7 (4.6)	5 (8.1)	2 (2.2)	0.12
Pillow, n (%)	22 (14.5)	13 (21.0)	9 (10.0)	0.07
Extra blanket, n (%)	91 (59.9)	51 (82.3)	40 (44.4)	< 0.01
Suction, n (%)	3 (2.0)	3 (4.8)	0 (0.0)	0.07
Fluffy blanket, n (%)	53 (34.9)	22 (35.5)	31 (34.4)	1.0
Med equipment NOT in use, n (%)	18 (11.8)	13 (21.0)	5 (5.6)	< 0.01
Other loose, n (%)	59 (38.8)	40 (64.5)	19 (21.1)	< 0.01

**Table 3:** Evaluation of ABC Compliance pre and post intervention

Variable	Overall (*n* = 154)	Pre (*n* = 64)	Post(n = 90)	*p*-value
ABC Compliance^a^, n (%)	n, (%)	n, (%)	n, (%)	< 0.01
No	119 (78.3)	60 (96.8)	59 (65.6)	
Yes	33 (21.7)	2 (3.2)	31 (34.4)	

^a^ABC compliance: compliance with both safe sleep position and environment

### Crib card use and safe sleep adherence

In addition to the tracking boards and crib audits, some cribs (16/90, 17.8%) had signs placed on them in the post intervention period to reinforce the safe sleeping and environment. Cribs with a crib card were 25% compliant with ABC recommendations, while cribs without cards were 36.5% compliant with ABC recommendations (*p* = 0.56) (Table 4). Based on this evidence, we cannot conclude that in the post-intervention period signs significantly affected ABC compliance.

**Table 4:** Evaluation of crib cards in association with sleep position, sleep environment and overall ABC compliance

Variable	Overall (*n* = 90)	No card (*n* = 74)	Crib card present (*n* = 16)	*p*-value
Safe sleep position, n (%)	n, (%)	n, (%)	n, (%)	0.29
Not safe	16 (17.8)	15 (20.3)	1 (6.2)	
Safe	74 (82.2)	59 (79.7)	15 (93.8)	
Safe environment, n (%)				0.39
Not safe	56 (62.2)	44 (59.5)	12 (75.0)	
Safe	34 (37.8)	30 (40.5)	4 (25.0)	
ABC Compliance, n (%)				0.56
Not safe	59 (65.6)	47 (63.5)	12 (75.0)	

### Nurse survey results

A post-intervention survey was sent electronically to 141 nurses who worked on the two general pediatric floors that participated in our study, with 75 nurses responding, giving a response rate of 53.2%. Most nurses 61.3% (*n* = 46) stated that parents putting items in a crib made it a challenge to ensure a safe sleep environment, while 22.7% (*n* = 17) of nurses reported the challenge as infants requiring more intervention due to crying or vital sign changes with supine (back) positioning. Fewer nurses 5.3% (*n* = 4) replied not feeling comfortable proving education as the challenge to providing a safe sleep environment, and 12% (*n* = 9) said “other.” As for the helpfulness of safe sleep signs, 64% (*n* = 48) of nurses stated the signs were “not at all helpful” or “a little helpful,” while 22.7% (*n* = 17) stated “moderately helpful.” Only 14.7% (*n* = 11) of nurses reported the safe sleep signs as “very helpful” or “extremely helpful.”

## Discussion

In this study of a safe infant sleep QI program to improve adherence to AAP safe sleep recommendations in hospitalized infants, the overall compliance with ABC recommendations improved significantly post-intervention. Sleep position did not significantly change due to a high number of correct baseline sleep positioning. The high number of baseline back/supine sleeping is expected due to the Back to Sleep campaign and the length of time the recommendation has been in place, as has been supported by other QI programs. (Shapiro-Mendoza, 2017; de Luca & Hinde, 2016; Colson et al., 2017; Kuhlmann et al., 2016)

When evaluating sleep position in our study, the majority of infants were safe; however, close to 20% of infants were still in unsafe sleep positions. The most common unsafe sleep position was prone. Despite overall improvement in supine positioning over the past 20 years, some recent studies have shown a stagnant number of infants again sleeping prone and that healthcare providers are not communicating the risks of prone sleeping, which our study supports. (Moon, 2016; de Luca & Hinde, 2016; Colson et al., 2017) Our results support the need to continue to educate about risks of prone sleeping.

There were also a number of infants asleep in a caregiver’s bed. This may be due to the ability of caregivers to sign a waiver that allows co-sleeping when inpatient in our pediatric facility. Additionally, the desire to comfort infants during times of illness and hospitalization may also lead to more co-sleeping than expected. Ideally in the future, we would hope to remove the co-sleeping waiver at our facility.

The sleep environment improved with fewer nonessential items observed in the infant’s crib after program implementation. Due to significant improvement in the sleep environment, there was a significant improvement in overall ABC compliance.

Objects that can cause suffocation, including soft objects, loose bedding and pillows, can obstruct airways and increase risk of SIDS and ASSB. (Moon, 2016; Shapiro-Mendoza et al., 2015; Sheers et al., 1998; Patel & Harris K Thach, 2001; Kemp et al., 1998; Kanetake et al., 2003) Previous studies have also shown low baseline numbers of safe sleep environments in hospitals, supporting that although great strides have been made in promoting “back to sleep”, continued work is needed on promoting the “Alone” of the ABCs of safe sleep. (Kuhlmann et al., 2016; Shadman et al., 2016; Macklin et al., 2016) Storage bins have been used in other studies along with sleep sacks to assist with removal of items and extra blankets from the cribs and may be implemented in the future as we continue our safe sleep work. (Kuhlmann et al., 2016; Zachritz et al., 2016; McMullen et al., 2016)

Although we saw change in compliance with ABC recommendations, we could not make conclusions about the effectiveness of the crib cards due to low compliance of use. Previous studies have demonstrated significant improvement in following ABC compliance with crib cards (Hwang et al., 2015; Gelfer et al., 2013) Interestingly, our study findings are validated with our nurse survey results that showed a majority of nurses did not find crib cards to be very helpful. It is possible that crib cards were more readily placed on cribs of infants with parents who were resistant to safe sleep measures.

Previous studies have also shown that nurse attitudes took time to change and did not improve as much with short safe sleep interventions as expected. (McMullen et al., 2016) Because crib cards were not permanent fixtures on the cribs, they were removed during cleaning, and often not replaced when an infant < 12 months was roomed. Thus many were lost over time. By the end of our study period, there were very few crib cards left. Because lack of crib cards was not communicated to nurse administrators, more crib cards were not made available. Perhaps if the crib cards had been permanently affixed to cribs, there would have been better compliance and stronger impact. This raises the question about what other factors promoted the change in safe sleep habits in this tertiary hospital setting. It is suspected that the Hawthorne effect may be at play, and the changes in compliance may have occurred because hospital staff was aware of the intervention and crib audit observations.

### Limitations

Data were collected by limited convenience sample over a relatively short amount of time (with post-intervention only occurring over a four-week period) with a relatively short follow-up period. Also, we did not compare floors with the QI program to floors without, although we did conduct baseline data collection before implementation of our safe sleep initiative. Because data sampling was not always performed at the same time daily, there was a sampling bias based on time of audits. Although there was an attempt to vary times, the results may have varied depending on times that the audits were performed. Because the tracking boards were placed in the nurse breakroom, it is unclear if all nurses involved saw the tracking boards on a weekly basis. There were a limited number of crib cards and poor compliance with placement of the crib cards; therefore, it is difficult to tell the impact of the crib cards. The study was only performed at a single site, so generalizability of the data is unclear. However, there are future plans to expand this study to involve two general pediatric floors at another campus of our pediatric hospital.

## Conclusions

A QI program utilizing crib cards, tracking boards, nursing education, and crib audits improved adherence to the AAP’s ABC’s of infant safe sleep of hospitalized infants. This intervention had a larger impact on sleep environment compared to sleep position. However, crib cards alone did not play a significant role in this change. Future research, training and education are necessary to understand what factors caused the improvement in compliance, to identify what additional educational materials are needed and to evaluate additional barriers to safe infant sleep in the tertiary care setting.

## Abbreviations

AAP: American Academy of Pediatrics
ABC: Alone, on back and in crib
ASSB: Accidental suffocation and strangulation in bed
DPH: Department of public health
IRB: Institutional review board
QI: Quality improvement
SUID: Sudden unexplained infant death
US: United States
